# Precedent Fluctuation of Serum hs-CRP to Albumin Ratios and Mortality Risk of Clinically Stable Hemodialysis Patients

**DOI:** 10.1371/journal.pone.0120266

**Published:** 2015-03-20

**Authors:** Jyh-Chang Hwang, Ming-Yan Jiang, Yi-Hua Lu, Charn-Ting Wang

**Affiliations:** 1 Division of Nephrology, Chi Mei Medical Center, Tainan, Taiwan; 2 Department of Hospital and Health Care Administration, Chia Nan University of Pharmacy and Science, Tainan, Taiwan; Sao Paulo State University, BRAZIL

## Abstract

**Background:**

A high sensitivity C-reactive protein to albumin ratio (hs-CRP/Alb) predicts mortality risk in patients with acute kidney injury. However, it varies dynamically. This study was conducted to evaluate whether a variation of this marker was associated with long-term outcome in clinically stable hemodialysis (HD) patients.

**Methods:**

hs-CRP/Alb was checked bimonthly in 284 clinically stable HD outpatients throughout all of 2008. Based on the “slope” of trend equation derived from 5–6 hs-CRP/alb ratios for each patient, the total number of patients was divided into quartiles—Group 1: β≦ −0.13, n = 71; group 2: β>-0.13≦0.003; n = 71, group 3: β>0.003≦0.20; and group 4: β>0.20, n = 71. The observation period was from January 1, 2009 to August 31, 2012.

**Results:**

Group 1+4 showed a worse long-term survival (p = 0.04) and a longer 5-year hospitalization stay than Group 2+3 (38.7±44.4 vs. 16.7±22.4 days, p<0.001). Group 1+4 were associated with older age (OR = 1.03, 95% CI = 1.01–1.05) and a high prevalence of congestive heart failure (OR = 2.02, 95% CI = 1.00–4.11). Standard deviation (SD) of hs-CRP/Alb was associated with male sex (β = 0.17, p = 0.003), higher Davies co-morbidity score (β = 0.16, p = 0.03), and baseline hs-CRP (β = 0.39, p<0.001). Patients with lower baseline and stable trend of hs-CRP/Alb had a better prognosis. By multivariate Cox proportional methods, SD of hs-CRP/alb (HR: 1.05, 95% CI: 1.01–1.08) rather than baseline hs-CRP/Alb was an independent predictive factor for long-term mortality after adjusting for sex and HD vintage.

**Conclusion:**

Clinically stable HD patients with a fluctuating variation of hs-CRP/Alb are characterized by old age, and more co-morbidity, and they tend to have longer subsequent hospitalization stay and higher mortality risk.

## Introduction

Patients with end-stage renal disease (ESRD) carry various inflammatory burdens, which play a pivotal role in leading to their wasting and malnutrition status. Thus, wasting in ESRD patients was strongly associated with a persistent systemic inflammation, cardiovascular disorders, and early death [1]. Several markers, such as high sensitivity C-reactive protein (hs-CRP), interleukin (IL)-6, IL-8, and serum albumin levels (S[Alb]), were frequently applied to evaluate malnutrition, inflammation and atherosclerosis (MIA) syndrome in chronic dialysis patients [2,3].

Owing to the fact that they are readily accessible and regularly monitored, S[Alb] and hs-CRP were most prevalently monitored in clinical practice. Hypoalbuminemia has long been recognized as a strong predictor of a worse long-term prognosis in chronic kidney disease (CKD) patients [4]. hs-CRP was associated with the risks of coronary heart disease, ischemic stroke, and cardiovascular mortality in general population [5]. It is also related to all-cause and cardiovascular death in ESRD patients [6]. Thus, the integration of both, namely, utilizing the hs-CRP to S[Alb] ratio (hs-CRP/Alb), has more value than either alone with regards to making predictions of the prognosis of patients in intensive care units [7]. A high ratio of hs-CRP/Alb indicates higher residual inflammation superimposed with malnutrition status. This marker has rarely been employed in the study of ESRD patients on chronic hemodialysis (HD).

Most studies with regards to monitoring ESRD patients’ survival were based on the single data of the inflammatory index [4,6]. In fact, these marker levels would undulate dynamically over time according to the repetitive occurrence and subsidence of inflammatory events. Few studies assessed the “trends” and “sequential changes” of these markers in screening ESRD patients’ long-term outcomes [8]. Recently, we found that progressively increasing hs-CRP levels were associated with subsequent peritonitis episodes in chronic ambulatory peritoneal dialysis patients [9]. This indicates that MIA syndrome may occur at any moment in uremic patients with an ongoing subtle infection/inflammation episode without any clinical manifestation.

Therefore, in an effort to elucidate the “trend" of inflammatory markers and its association with the long-term outcomes of HD patients, we identified several questions which need to be answered. Do clinically stable ESRD outpatients maintain a fixed hs-CRP/Alb ratio? Is baseline or fluctuating hs-CRP/Alb ratio a more powerful long-term mortality predictor for chronic HD patients? Is there any survival difference between chronic HD patients with progressive increase or decrease in hs-CRP/Alb? Is fluctuating hs-CRP/Alb ratio a negative factor in evaluating the morbidity and mortality risks of chronic HD patients? At present, there is no clear consensus on the answers to these questions. Therefore, this study was conducted to feature those clinically stable HD outpatients with variously fluctuating hs-CRP/Alb ratios, and also to compare the co-morbidities, the subsequent length of hospitalization stay, and the association of long-term mortality of chronic HD patients with the various ratios of hs-CRP/Alb.

## Patients and Methods

In February 2008, i.e., year 0, a total of 407 ESRD outpatients received regular in-center HD through either native arteriovenous fistula or polytetrafluoroethylene graft, three times a week, 4 hours a session, at the Chi Mei Medical Center. The exclusion criteria were patients shifting to peritoneal dialysis (n = 2), using central venous catheters for temporary dialysis, suffering acute kidney injury and transferred to other HD centers (n = 78), receiving renal transplantation (n = 2), those patients who were deceased or hospitalized for various etiologies during the period of regular monthly blood check from February 2008 to December 2008 (n = 39), and those who had missed data with less than 5 in the number of hs-CRP /Alb ratio (n = 2) during the year 0. Those data of hs-CRP and S[Alb] obtained during the hospitalization period or when patients visited the emergency department were excluded. The inclusion criteria were that patients received regular HD at our unit for more than 6 months and had 5 or more hs-CRP/Alb ratios in the year 0. The observation period was from January 1, 2009 to July 31, 2012, i.e., year 1 to year 3.

All pre-HD biochemical data including S[Alb] (bromocresol purple method), potassium, urea nitrogen (BUN), creatinine, uric acid and phosphate concentrations were measured by Hitachi 7601–110 Automatic Analyzer (Tokyo, Japan) on the last Wednesday and/or Thursday of each month, while hs-CRP (CardioPhase Siemens Healthcare Diagnostics Products, GmbH, Germany) was additionally checked bimonthly from February to December during the year 0. The ratio of hs-CRP/Alb was calculated for each patient by a quotient of hs-CRP divided by S[Alb]. Hematocrit was measured by Beckman Coulter LH755-A (Fullerton, California). We also evaluated urea reduction rate and normalized protein catabolism (nPCR) [10] for all patients at the beginning of study to compare the difference in dialysis dosage and protein intake status between groups. The clinical records and data of patients used in this study were anonymized and de-identified prior to analysis.

The hollow-fiber dialyzers applied to all patients during the study period included DICEA-210G (Baxter Healthcare Corporation. IL, USA), AM-BIO-HX-100, FASFLO-PS-18H (Ashahi kasei Kuraray Medical Co. Tokyo, Japan), Hemoflow F60, F80 (Fresenius Medical Care AG. Bad, Homburn), and FB-210A (Nisso Corporation. Osaka, Japan). The formula of the dialysate bath used in the study was sodium: 139.0 mEq/L, calcium: 3.0 mEq/L, potassium: 2.0 mEq/L, magnesium: 1.0 mEq/L, chloride: 106.5mEq/L, acetate: 4.0mEq/L, dextrose: 200mg/dL, and bicarbonate: 39mEq/L (Hemodialysis Concentrate A-35 & BP-11, Chi Sheng Chemical Corporation, Hsinchu, Taiwan). Hospitalization days of each patient were monitored and recorded yearly from January 1, 2007 (i.e., year −1) to the end of study, i.e., July 31, 2012. The co-morbidity factors were determined by Davies co-morbidity scores [11] according to the past history recorded in the medical charts at the beginning of study.

## Statistical Analysis

Appropriate χ^2^ and ANOVA with Bonferroni tests were used for comparisons between categorical and continuous variables respectively between the groups, as shown in Table 1. The "slope of trend equation" was calculated by Microsoft Excel 2010. The relationship between fluctuation changes (standard deviation, SD) of hs-CRP and SD of S[Alb] was assessed by Pearson correlation test. In total, 1,686 pairs of hs-CRP and S[Alb] were collected during the year 0 for all 284 patients to evaluate the reciprocal inter-relationship. Differences in the length of hospitalization between Group 1+4 vs. Group 2+3 of each year and total 5 years were evaluated by Mann-Whitney U tests. Actuarial survival rates of the different groups were determined by the Kaplan-Meier methods, and log rank tests were employed to compare the different survival curves between each group. Multivariate logistic regressions were applied to analyze the independent clinical factors associated with the group with fluctuating hs-CRP/Alb ratios (i.e., Group 1+4). In addition, multivariate Cox proportional hazard methods were performed to compare the hazard ratio between SD and baseline hs-CRP/Alb in the long-term mortality risk. All data are expressed as mean ± SD. A *p* value of less than 0.05 was considered to be significant. Computations were performed with the SPSS 17.0 package for Windows (SPSS, Chicago, IL).

**Table 1:** Basic demographic differences among the 4 groups in the study.

	Group 1	Group 2	Group 3	Group 4
	(n = 71)	(n = 71)	(n = 71)	(n = 71)
**Demographic factors**				
Mean age at start of study, years	62±13	57±13^¶¶^	60±13	65±12
(range)	(30–84)	(22–86)	(38–91)	(32–86)
Male, %	41	51	49	48
Diabetes, %	32^a^	20^aa^	38^#^	51
Hemodialysis vintage, months	74±53	73±59	72±49	62±44
Ultrafiltration rate, L/session	2.7±1.2	2.7±0.9	2.9±0.9	2.8±0.9
Urea reduction rate, %	73.4±7.6	73.4±6.3	74.1±6.9	73.3±6.5
nPCR, g/day	1.11±0.27	1.21±0.31	1.15±0.27	1.14±0.29
Pre-HD mean BP, mmHg	100±16	100±17	101±14	102±18
CCr, mL/min	5.8±1.8	5.7±1.4	5.7±1.5	6.0±1.9
**Clinical comorbidity, %**				
Malignancy	17	11	17	15
Coronary artery disease	34	25	35	42
Peripheral vascular disease	24	15	24	38
Congestive heart failure	20	13	10	25
Others	7	4	8	8
Davies score	1.4±1.2	0.9±1.2^¶¶^	1.4±1.3	1.9±1.3
**Laboratory data**				
Slope of trend equation (range)	−1.01±1.32 (−5.81 to −0.13)	−0.05±1.32** ^¶¶^ (−0.13 to 0.003)	0.07±0.06** ^¶¶^ (0.003 to 0.20)	1.05±1.56** (0.20 to 0.49)
Variation of hs-CRP to albumin ratio*	4.25±5.66	0.45±0.50** ^¶¶^	0.53±0.54** ^¶¶^	4.30±5.80
Albumin, g/dL	3.7±0.5	4.0±0.3**	4.1±0.3** ^¶^	3.9±0.4
hs-CRP, mg/L	20.5±26.3	4.0±5.2**	3.2±4.4**	8.7±9.6**
HgA1C, %	6.3±1.6	5.9±1.4^¶^	6.3±1.7	6.8±2.0
BUN, mg/dL	67±17	73±18	69±16	71±19
Creatinine, mg/dL	9.7±2.4	10.7±2.4	10.4±2.5	9.7±2.6
Sodium, mEq/L	137.7±2.9	138.7±2.8	139.4±3.4*	138.7±2.8
Potassium, mEq/L	4.5±1.0	4.7±0.8	4.8±0.8	4.9±1.0
Calcium, mg/dL	8.9±0.8	9.2±0.8	9.1±0.7	9.1±0.7
Phosphate, mg/dL	5.1±1.6	5.4±1.5	5.0±1.4	5.2±1.4
Total cholesterol, mg/dL	186±44	196±37	181±36	179±42
Hemoglobin, mg/dL	9.0±1.8	10.0±1.6*	10.0±1.3*	9.7±1.4*

Abbreviations: BW = body weight, nPCR = normalized protein catabolism rate, HD = hemodialysis, BUN = blood urea nitrogen, SD = standard deviation, hs-CRP = high sensitivity C-reactive protein, HgA1C = Hemoglobin A1C

*: standard deviation of hs-CRP to albumin ratios

Statistics:

*:p<0.05, **: p<0.001 vs. group 1 (ANOVA with Bonferroni tests)

^§^: p<0.05, ^§§^: p<0.001 vs. group 2 (ANOVA with Bonferroni tests)

^¶^: p<0.05, ^¶¶^: p<0.001 vs. group 4 (ANOVA with Bonferroni tests)

^#^:p<0.05, vs. group 2 (Chi-square tests)

^a^:p<0.05, ^aa^: p<0.001 vs. group 4 (Chi-square tests)

## Results

### Baseline demographic characteristics of patients

**Fig 1:**
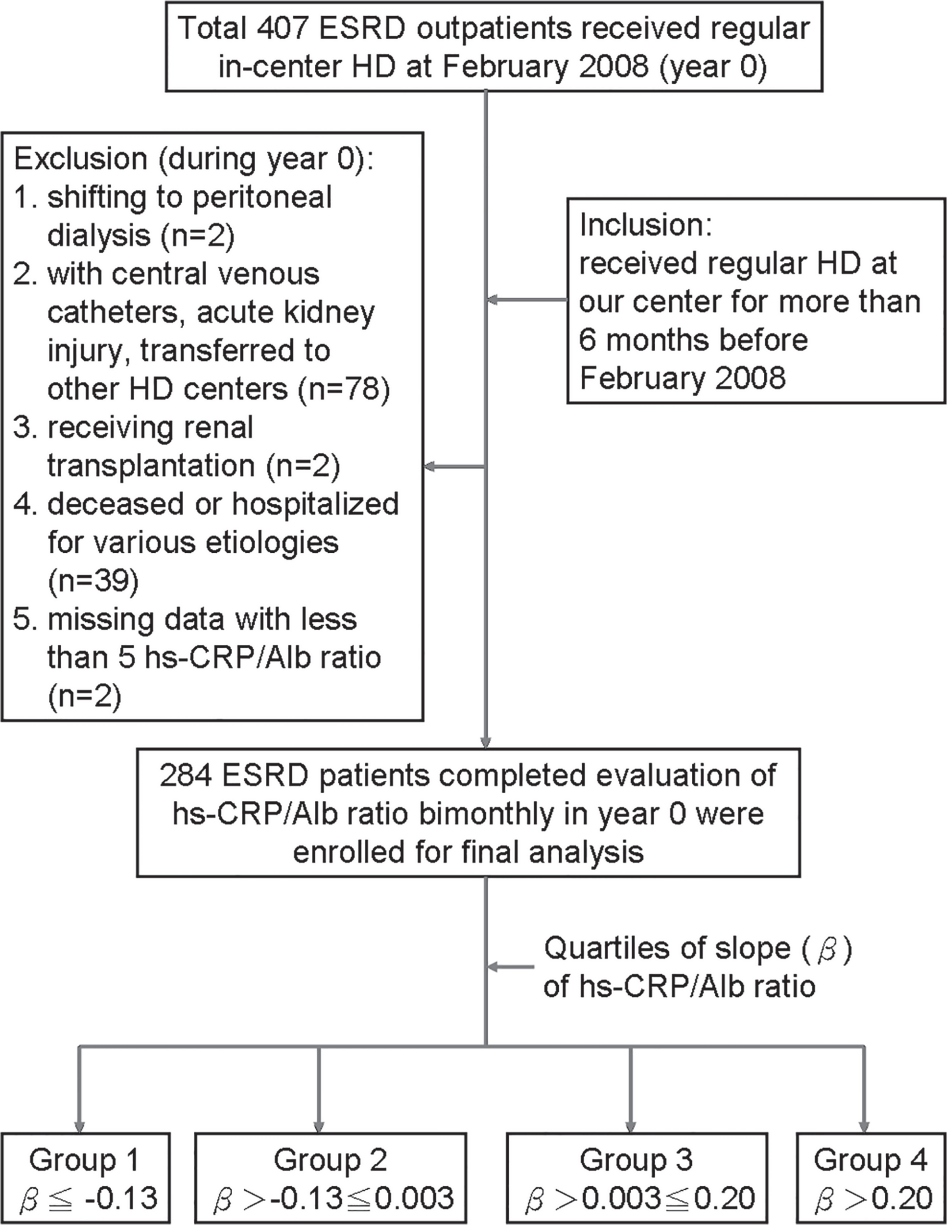
Study flowchart (ESRD: end-stage renal disease, HD: hemodialysis, hs-CRP: high sensitivity C reactive protein, Alb: albumin).

A total of 284 ESRD patients on HD (mean age at the initiation of year 0: 61 ± 13 years; range: 22–91 years; male: 47%) fulfilled the criteria and were enrolled in this prospective cohort study. The renal etiologies were attributed to chronic glomerulonephritis in 41% of patients, diabetic nephropathy in 36%, chronic tubulointerstitial nephritis in 8%, hypertensive nephrosclerosis in 6%, and other etiologies in the remaining 9%. A “trend equation” was deduced from all the ratios of hs-CRP/Alb checked in the year 0 for each patient. According to the “slope” (β) of the trend equation, all the patients were divided into quartiles—Group 1: β≦−0.13; n = 71; Group 2: β>−0.13≦0.003; n = 71; Group 3: β>0.003≦0.20; n = 71, and Group 4: β>0.20, n = 71 (Fig. 1). Compared to their counterparts, Groups 1 and 4, with higher absolute values in the slope of hs-CRP/Alb, were characterized by more fluctuating hs-CRP/Alb ratios. All the slopes of total clinically stable HD patients were presented as a normal distribution, with nearly half (44.8%) ranging between −0.125 and +0.125.

As shown in Table 1, compared to Groups 2 and 3, Group 4 had a higher prevalence of diabetes mellitus, higher Davies co-morbidity score, and older age. Groups 1 and 4 also had higher variation of hs-CRP/Alb ratios, and lower baseline S[Alb] compared to Groups 2 and 3. There was no significant difference in SD of hs-CRP/Alb ratio between Groups 1 and 4, nor any difference between Groups 2 and 3. Group 1 had the highest baseline hs-CRP and lowest hemoglobin level.

### The characteristics of patients with fluctuating hs-CRP to albumin ratios (i.e., group 1+4)

Multivariate logistic regression tests were applied to study the characteristics of Group 1+4, since these patients tended to have higher hospitalization rates than their counterparts. Group 1+4 were found to be associated with older age (OR = 1.03, 95%CI = 1.01–1.05), and a high prevalence of congestive heart failure (OR = 2.02, 95%CI = 1.00–4.11) (Table 2). By multiple linear regression tests, SD of hs-CRP/Alb was significantly correlated to male gender, higher baseline hs-CRP level and Davies co-morbidity score (Table 3).

**Table 2:** Multivariate logistic regression tests for evaluation the characteristics of patients with highly fluctuated high sensitive C-reactive protein to albumin ratios (i.e., Group 1+4).

		95% CI for OR		
	OR	Lower	Upper	p
Male gender, y or n	0.88	0.55	1.42	0.63
Diabetes, y or n	1.60	0.95	2.70	0.08
Age*, year	1.03	1.01	1.05	0.009
malignancy, y or n	1.05	0.54	2.07	0.88
Coronary artery disease, y or n	0.90	0.51	1.59	0.71
Congestive heart failure, y or n	2.02	1.00	4.11	0.05

Dependent variable: Group 1+4 (vs. Group 2+3);

* age at the initiation of study

**Table 3:** Risk factors associated with afluctuation of high sensitivity C-reactive protein to albumin ratio evaluated by multiple linear regression tests.

		95%CI		
	B	Lower	Upper	p
Male gender, y or n	0.17	0.54	2.53	0.003
Davies scores	0.18	0.06	1.16	0.03
Baseline hs-CRP, mg/L	0.39	0.08	0.14	<0.001
Diabetes, y or n	0.03	−1.15	1.63	0.73
Hemodialysis vintage, month	0.90	0.00	0.02	0.10
Baseline serum albumin, g/dL	−0.10	−2.52	0.31	0.13

^a^. Dependent variable: standard deviation of hs-CRP/Albumin ratio

Abbreviation: hs-CRP = high sensitive C-reactive protein

### Correlation of hs-CRP fluctuation and changes of serum albumin concentrations

**Fig 2:**
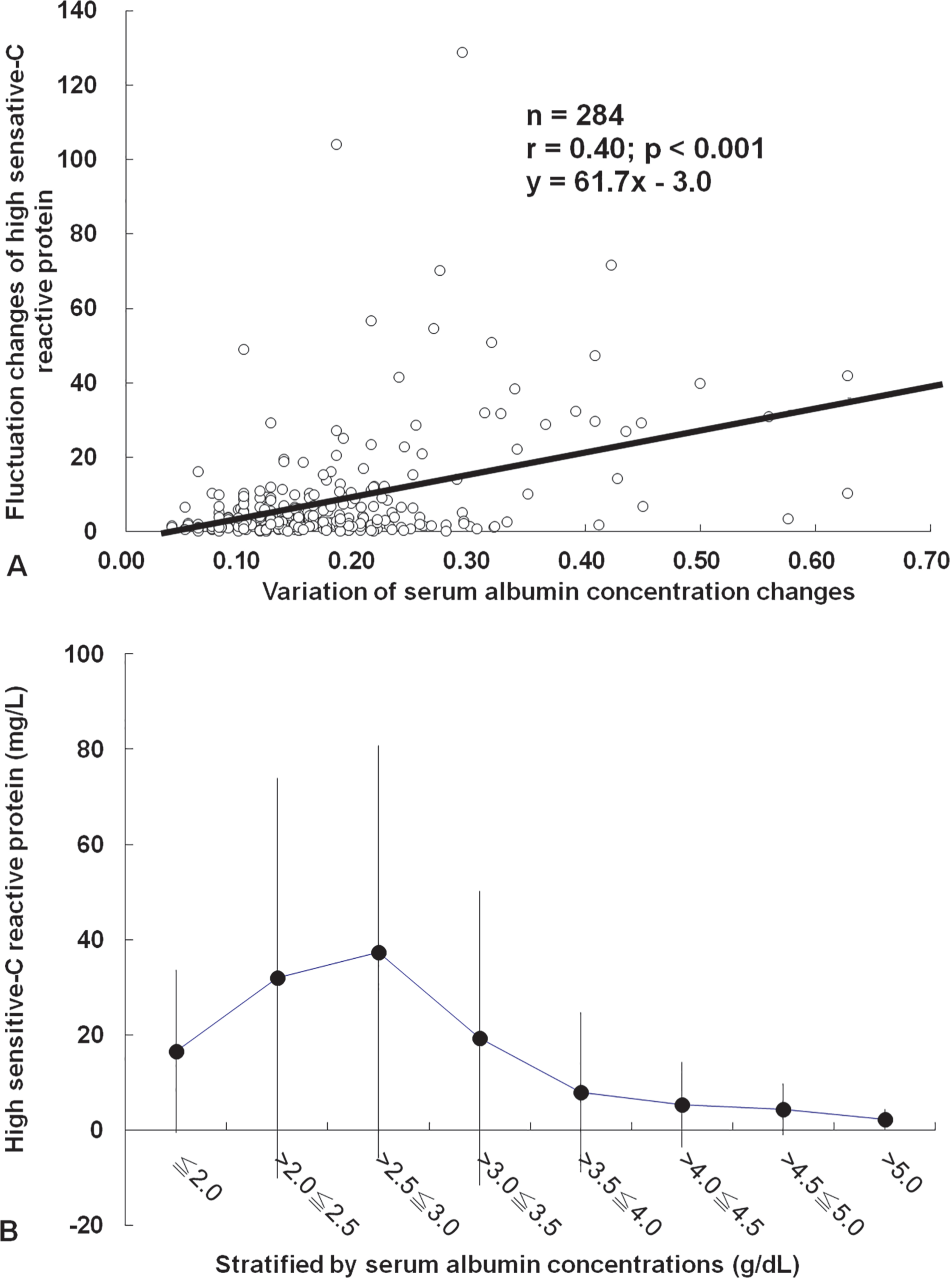
Correlation of hs-CRP fluctuation and changes of serum albumin concentrations (A) Fluctuation (standard deviation, SD) of hs-CRP changes was positively correlated to variation (SD) of serum albumin concentrations. (B) hs-CRP decline significantly only if S[Alb] was higher than 3.5g/dL.

SD of hs-CRP was inversely correlated to the SD of S[Alb] (r = −0.40; p<0.001, Fig. 2A). As shown in Fig. 2B, hs-CRP declined significantly only if S[Alb] was greater than 3.5g/dL.

### Difference of hospitalization days between Group 2+3 vs. 1+4 over the 5 years

**Fig 3:**
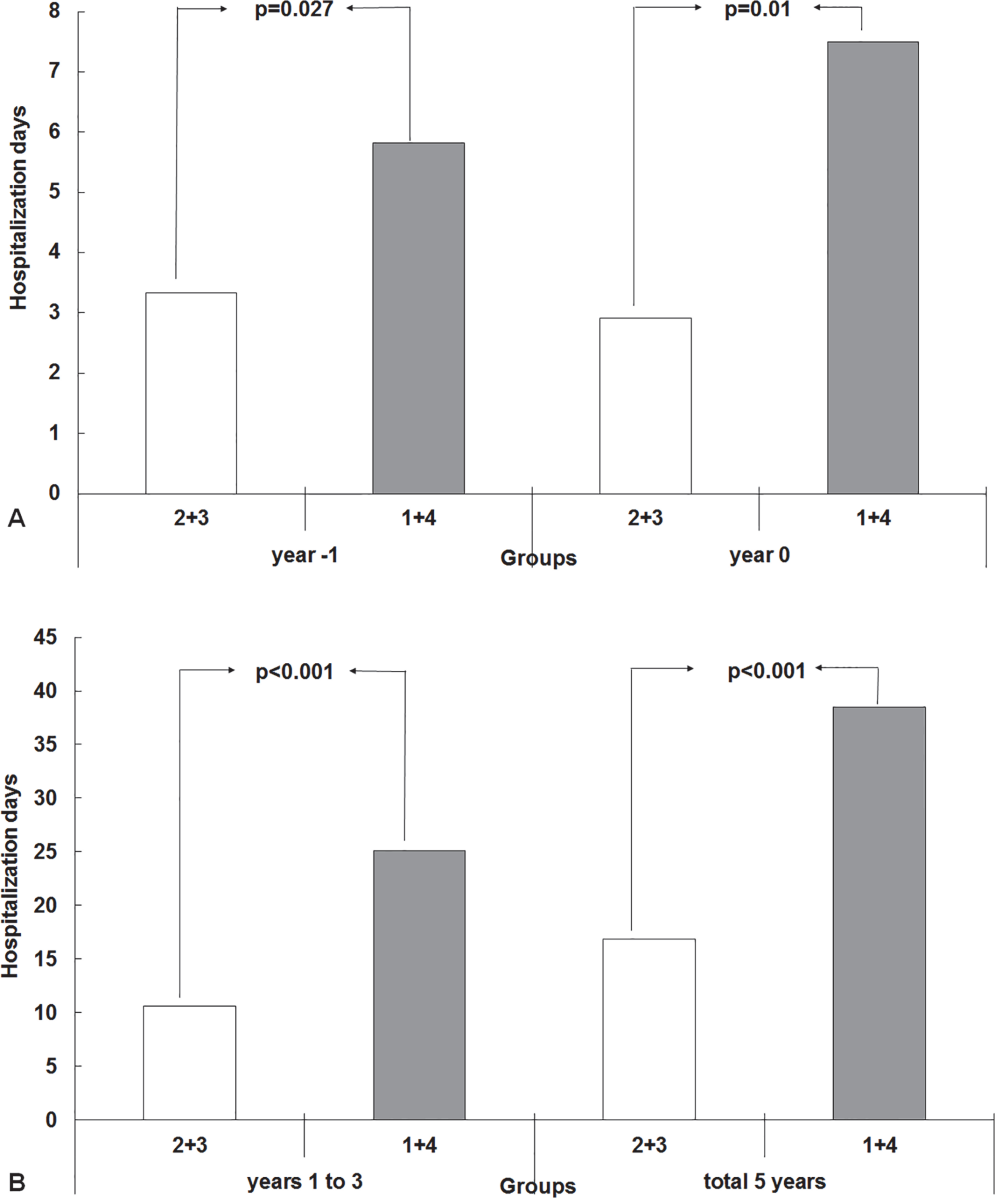
Difference of hospitalization days between Group 2+3 vs. 1+4 over the 5 years (A) Compared to the Group 2+3, Group 1+4 showed higher hospitalization length in one year before evaluation (year −1 = 5.9±11.0 vs. 3.3±7.9 days, p = 0.02), and year 0 (7.6±15.2 vs. 2.9±19,8 days, p = 0.003). (B) Group 1+4 also had longer hospitalization stay in the subsequent 3 years (years 1 to 3 = 25.3±35.6 vs. 10.6±15.0 days, p<0.001) and total 5 years (38.7±44.4 vs. 16.7±22.4 days, p<0.001).

Compared to the Group 2+3, Group 1+4 showed longer hospitalization length in the year before evaluation (year −1 = 5.9±11.0 vs. 3.3±7.9 days, p = 0.02), and the year 0 (7.6±15.2 vs. 2.9±19,8 days, p = 0.003, Fig. 3A). Group 1+4 also had longer hospitalization stays in the subsequent 3 years (years 1 to 3 = 25.3±35.6 vs. 10.6±15.0 days, p<0.001) and total 5 years (38.7±44.4 vs. 16.7±22.4 days, p<0.001, Fig. 3B).

### Survival differences among 4 groups

**Fig 4:**
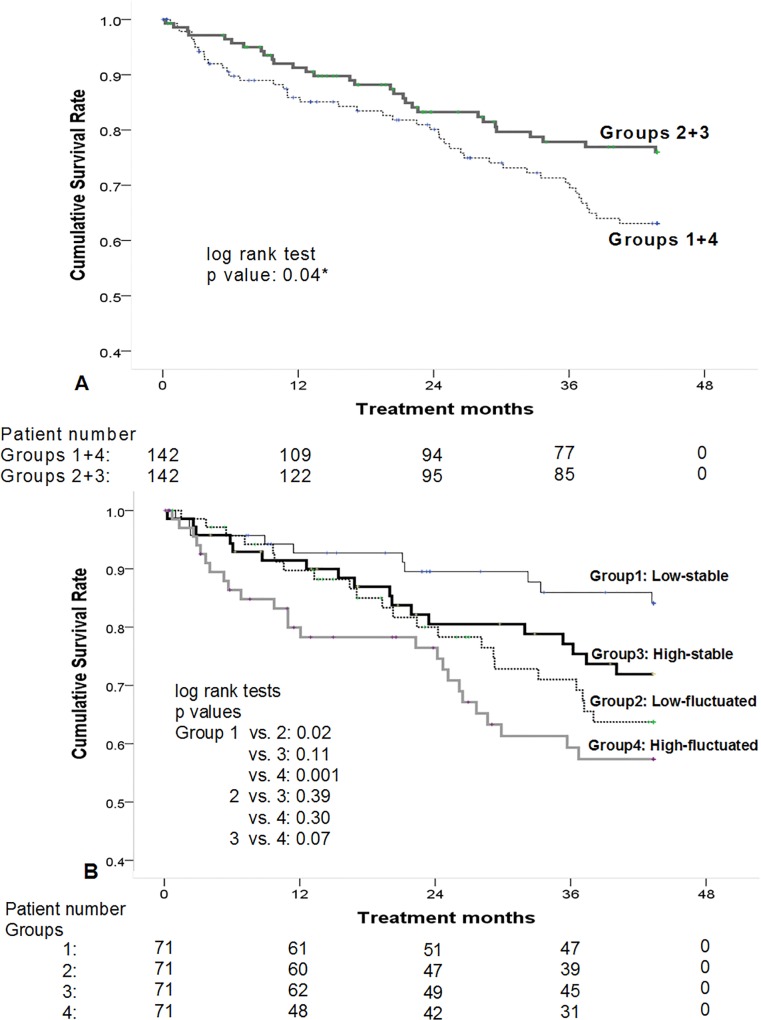
Survival differences among 4 groups (A) By Kaplan-Meier analyses, Group 2+3 showed a higher cumulative survival rate than those with high fluctuation (i.e., Groups 1+4) (p = 0.04). (B) According to the medians of baseline hs-CRP/Alb and standard deviation of hs-CRP/Alb, total patients were re-divided into four groups. Low-stable group had a better survival than the low-fluctuated (p = 0.02), and high-fluctuated groups (p = 0.001). However, there were no significant survival differences between the high-stable, low-fluctuated, and high-fluctuated groups.

After utilizing Kaplan-Meier analyses, Group 2+3 showed a higher cumulative survival rate than those with high fluctuation of hs-CRP/Alb (i.e., Group 1+4) (p = 0.04, Fig. 4A). There was no difference in long-term outcomes between Groups 1+2 and 3+4 (p = 0.29). After being divided into 4 groups, Group 2 had a better outcome than Groups 1 and 4, but there was no difference between Groups 2 and 3 in cumulative survival (p = 0.11), or between Groups 1 and 4 (p = 0.97). For evaluation of the combined impact of baseline hs-CRP/Alb and fluctuation of hs-CRP/Alb on the long-term prognosis of chronic HD patients, total patients (n = 284) were divided into “high” (n = 142) and “low” (n = 142) groups based on the median baseline hs-CRP/Alb (data of February 2008). By individual group’s median SD of hs-CRP/Alb, each group was further sub-divided into “fluctuated” and “stable” groups. As shown in Fig. 4B, low-stable group showed the highest cumulative survival rate, while high-fluctuated group revealed the worst outcomes. The low-stable group had a better survival than the low-fluctuated (p = 0.02) and high-fluctuated groups (p = 0.001). However, there were no significant survival differences between the high-stable, low-fluctuated, and high-fluctuated groups.

### Independent factors associated with long-term mortality

Since fluctuation of hs-CRP/Alb was associated with older age and co-morbid condition, these two covariates were removed from model 2 multivariate Cox proportional analysis (Table 4). After adjusting by HD vintage, gender, and diabetes, SD of hs-CRP/Alb rather than baseline hs-CRP/Alb was an independent factor associated with long-term mortality.

**Table 4:** Independent factors associated with mortality evaluated by multivariate Cox proportional methods.

	Model 1				Model 2			
	HR	95%CI		p	HR	95%CI		p
Variation of hs-CRP/albumin ratio*	1.02	0.98	1.06	0.28	1.05	1.01	1.08	0.02
Baseline hs-CRP/albumin ratio	1.03	0.99	1.07	0.16	1.03	0.99	1.07	0.11

* Standard deviation of hs-CRP/Albumin ratio

Abbreviations: hs-CRP = high sensitive C-reactive protein

Model 1: adjusted by hemodialysis vintage, gender, age at the start of study, and Davies co-morbidity score

Model 2: after withdrawal two co-variants “age at the start of study” and “Davies score” from model 1

## Discussion

This study was conducted to address the 3-year outcome prediction in chronic HD patients on the basis of the preceding one-year trend of hs-CRP/Alb ratio. The main finding was that the ratios of hs-CRP/Alb were normally distributed in these clinically stable patients. A previous fluctuation but not a progressive rise of this marker was an independent risk factor for long-term mortality. The amplification of hs-CRP elevation paralleled the extent of S[Alb] drop. Those patients with more fluctuating ratio changes were characterized by older age and greater prevalence of CHF. They also had more previous one-year and the succeeding 3 years’ of hospitalization days. Stability of hs-CRP/Alb ratio was definitely associated with better outcomes, especially in those patients with a lower baseline hs-CRP/Alb. Conversely, fluctuation of this ratio led to a higher mortality rate in both higher and lower baseline hs-CRP/Alb. Compared to the baseline hs-CRP/Alb ratio, a variation of the hs-CRP/Alb ratio has greater predictive power for long-term outcomes in HD patients under stable clinical conditions.

S[Alb] has been regarded as a marker of chronic malnutrition [12], and it was also considered as the most important negative acute phase protein [13]. Catabolism of Alb was directly correlated with the severity of acute infection [14]. On the other hand, high CRP level has an association with the risks of coronary heart disease, ischemic stroke, and death from various cancers and lung disease [5]. It also predicts all-cause and cardiovascular death in HD patients [6]. Although high hs-CRP and hypoalbuminemia were individual indicators as poor prognostic signs for CKD patients, the interaction between the two variables has been only rarely discussed [15]. Our data showed a reciprocal relationship between S[Alb] and hs-CRP levels. The extent of S[Alb] decline was inversely correlated to the degree of hs-CRP increase. Persistently high hs-CRP levels were found in S[Alb] with less than 3.5g/dL. Conversely, hs-CRP levels were continuously low if S[Alb] was higher than 4.0g/dL. This suggests that hypoalbuminemia is closely associated with the degrees of infection and inflammation. Combination effects of facilitated catabolism by infection/inflammation episodes, dilution from hypervolemia, and protein calorie malnutrition in HD patients lead to "hypoalbuminemia" [12].

However, why was the correlation coefficient (β) between the changes of these two variables only −0.40 in Fig. 2? We hypothesized that some unknown factors, such as co-existing different intensity of malnutrition, volume overload, various co-morbid status, and elapsed time by longer S[Alb] half-life, influenced the tightness of the inverse parallel between these two indices. From our data, these two markers still follow a divergent trend during or after the micro-infection and/or inflammation events even without apparent clinical manifestations.

Therefore, it is rational to combine hs-CRP and S[Alb]. We suppose the ratio of hs-CRP/Alb is a better surrogate marker than examining results individually to monitor MIA syndrome of chronic HD patients [16]. Prolonged or repeated hs-CRP/Alb ratio elevation, i.e., high hs-CRP with lower S[Alb], eventually leads to the PEW syndrome and poor long-term outcomes [17].

Based on the histogram hs-CRP/Alb ratio, the slopes of these patients were distributed normally. This signifies that even these clinically stable outpatients developed various degrees of subtle malnutrition/inflammations during the year 0, especially in Groups 1 and 4. These groups with fluctuating hs-CRP/Alb ratio were characterized by older age and higher prevalence of co-morbidities. The inherited demographic features predisposed these patients to develop a micro- infection/inflammation, which caused a discrepancy between hs-CRP and S[Alb]. The more the severe and frequent episodes that occurred, the more the ratio of hs-CRP/Alb fluctuated. Conversely, patients with flattened slopes, e.g., Group 2+3, tended to be under a more stable condition with very subtle change of hs-CRP, and S[Alb] in year 0. It was found in this study that both hs-CRP and S[Alb] of clinically stable patients varied dynamically over time. Stable trend of hs-CRP/Alb ratio was associated with a preferable prognosis, especially in the patients with lower initial hs-CRP/Alb ratios. However, those patents with high baseline and stable hs-CRP/Alb ratio had a similar outcome to the groups of fluctuating hs-CRP/Alb with either high or lower baseline data. Although a higher baseline hs-CRP/Alb was also a mortality risk, compared to the “spot data”, our data confirmed that the "fluctuation" of hs-CRP/Alb ratio was a stronger predictor for HD patients’ long-term outcome.

We applied the slope of hs-CRP/Alb ratio to reflect the dynamic status of MIA syndrome in chronic HD patients in year 0. Group 1+4 had a longer hospitalization stay in year −1. This indicated that residual infection/inflammation and the associated malnutrition developing during the year −1 led to a fluctuation of hs-CRP/Alb ratios in the following year. Group 1+4, of older age and with more co-morbid conditions, needed more medical care and longer hospitalization stay in the subsequent 3 years. Eventually, all these led to a high mortality.

Negative slope of hs-CRP/Alb ratio in Group 1+2 demonstrated a progressively decreasing inflammation/infection in the later half of year 0. However, it cannot be assumed that they will be clinically stable in the subsequent 3 years. Group 1+2, with a negative slope, had a similar long-term outcome compared to its positive-slope counterparts. This suggests that single data and an insufficient period of evaluation (less than 6 months) cannot accurately predict the subsequent 3-year prognosis of chronic HD patients.

This study has three strengths. First, we used the ratio of hs-CRP/Alb as an indicial marker to evaluate the clinical outcomes, since it integrates the effects of both inflammation and malnutrition. Second, this was the first time a study stratified patients on a basis of the “the slope of trend equations” of hs-CRP/Alb ratios over one year rather than using single data on a cross-sectional background. Slope of hs-CRP/Alb ratio trend seems to be a better parameter to monitor the ongoing illness-risk potentiality in clinically stable situations within a time period. We also proved sub-clinical inflammation/infection events developed even under a clinically “disease-free” status. Third, the hospitalization length of this study was extended to from one year before, and 3 years after the evaluation period. This provides more information about the causal relationship between the hs-CRP/Alb variability and clinical outcomes for a total of 5 years. However, there are also some weaknesses with regards to this study that should be considered. 30% of patients were withdrawn during year 0. Among them, one third with an inadequate number of hs-CRP/ratio ratios caused by death or hospitalizations due to major diseases was omitted. What would be the confounding effect had these data been included? However, these omissions were not expected to distort the estimate of long-term clinical outcomes of chronic HD patients with fluctuating changes of hs-CRP/Alb ratios.

In conclusion, evaluating the slope of hs-CRP/Alb ratio over a given time period offers a superior strategy for the risk stratification of HD patients. Clinically stable HD patients with preceding fluctuating hs-CRP/Alb ratios predicted worse subsequent 3-year outcomes. The fluctuations with regards to these ratio variations also led to a longer hospitalization stay. We hypothesized that these patients with more co-morbidity disorders were prone to be under frequent or repetitive micro-inflammation and malnutrition episodes, giving them a higher mortality risk. To predict the long-term prognoses of ESRD patients based on just one spot of either hs-CRP or S[Alb] would be misleading, since both are dynamically and unnoticeably changing. Although the variations of hs-CRP/Alb ratio are ignored in some clinically stable patients, they are strongly associated with subsequent morbidity and mortality. Combing baseline and trend of hs-CRP/Alb ratios will increase the accuracy in predicting the long-term outcomes of clinical stable HD patients. We recommend frequently monitoring the trend of this ratio on a regular basis in this group of patients.

### Ethical issues

The research ethics committee of the Chi Mei Medical Center has approved this study (IRB no. 10309-006).
